# Prevention of burnout syndrome in physicians: a systematic review and meta-analysis

**DOI:** 10.1007/s00508-025-02601-y

**Published:** 2025-11-10

**Authors:** Lea Krebs, Laura Jung, Jasmin Arrich

**Affiliations:** 1 Klinik für Unfall,- Hand- und Wiederherstellungschirurgie, Universitätsklinikum Jena, Am Klinikum 1, 07745 Jena, Germany; 2 Klinik für Notfallmedizin, Universitätsklinikum Jena, Am Klinikum 1, 07745 Jena, Germany; 3 Universitätsklinik für Notfallmedizin, Medical University of Vienna, Währinger Gürtel 18–20/6D, 1090 Wien, Austria

**Keywords:** Fatigue, Occupational health, Depression and anxiety, Stress, Intervention studies

## Abstract

**Background:**

Physicians are at high risk of developing burnout and several studies have evaluated burnout prevention programs. This meta-analysis and systematic review aimed to assess the effectiveness of burnout prevention programs and to evaluate whether one approach is superior to other programs.

**Patients, material and methods:**

The methods were based on the Cochrane Handbook of Systematic Reviews. We searched the literature in five medical databases from October 2019 to June 2022. We included randomized controlled trials that examined the effect of burnout prevention programs on physicians.

**Results:**

A total of 22 studies were included in our analysis, with 20 studies targeting individual interventions and 2 studies targeting structural interventions. Mindfulness-based stress reduction (MBSR) or similar programs were the most common interventions. The main analysis showed a significant reduction of burnout in the intervention groups with an standardised mean difference (SMD) of −0.32 (95% confidence interval, CI: −0.41 to −0.22), which suggests a small to moderate effect. MBSR did not appear superior to other interventions in the subgroup analysis SMD −0.25 (95% CI: −0.48 to −0.02) versus SMD −0.61 (95% CI: −1.19 to 0.03).

**Discussion:**

This analysis shows that individual burnout prevention programs may reduce burnout rates in physicians; however, the effect was relatively small. The effect was reduced even further when removing studies causing severe heterogeneity and those with a high risk of bias. Future research programs should focus on structural programs that address the lack of mentoring, rising administrative tasks, or long working hours, which may be more effective in reducing burnout in physicians.

**Supplementary Information:**

The online version of this article (10.1007/s00508-025-02601-y) contains supplementary material, which is available to authorized users.

## Introduction

The International Classification of Diseases 11 (ICD-11) describes burnout as a condition resulting from chronic stress at the workplace that has not been dealt with successfully. Burnout is often characterized by three symptoms: exhaustion or energy depletion, a negative attitude towards or mental distance from one’s job and reduced professional efficacy [1]. Burnout not only takes a toll on mental health but also significantly impacts physical well-being. It may be an independent risk factor for coronary heart disease, hypercholesterolemia, type 2 diabetes or headaches [2]. Prolonged burnout can lead to clinical depression [3], substance abuse and suicidal thoughts. For physicians, it can lead to an increased rate of professional errors and impaired communication with patients [4]. Poor quality of care, low patient satisfaction and compromised patient safety may be the consequences.

In the Medscape National Physician Burnout & Depression Report 2021, 12,000 physicians were asked about their mental health and 42% of them reported experiencing burnout [5]. Since the COVID-19 pandemic, reported burnout rates have continued to rise, particularly in countries with high numbers of severe cases of COVID-19 infections [6].

Burnout prevention programs can be categorized into two main groups: the individual approach, which targets physicians themselves and includes discussion groups incorporating elements of mindfulness, reflection, shared experience and small group learning [7], exercise interventions like low-intensity running sessions [8], swimming, walking, or indoor cycling [9] that have shown some effectiveness in decreasing emotional exhaustion in physicians. Organizational interventions are aimed at improvement of the work environment [10]. They may include work hour limitations [11], learning environment and workflow streamlining [12].

Physician burnout is a major issue in our healthcare system, with multiple ramifications on the well-being of the medical team and patients [13, 14]. Reliable evidence on effective prevention programs is essential to help clinicians and employers efficiently address this issue.

The objectives of this meta-analysis and systematic review were to assess the effectiveness of burnout prevention programs and to evaluate whether one approach is superior to other programs.

## Patients, material and methods

Methods and reporting of this systematic review and meta-analysis were based on the Cochrane Handbook for Systematic Reviews of Interventions Version 6 [15] and the Preferred Reporting Items for Systematic Reviews and Meta-analyses (PRISMA). We used EndNote (version 20.5.1) for the export, selection, and management of all studies. Data were entered into Review Manager 5 (The Nordic Cochrane Centre, Copenhagen, Denmark) and STATA (16.1, StataCorp LLC, College Station, TX, USA) and analyzed there. The study protocol was registered in PROSPERO (International Prospective Register of systematic reviews, CRD42022341538).

### Search strategy

The search terms included synonyms of “physician”, “burnout” and “prevention” and a filter for randomized controlled trials (see Supplementary Appendix 2 for the detailed search strategy). We searched MEDLINE Ovid, PsychINFO Ovid, BIOSIS Previews, SCOPUS and CENTRAL from inception to June 2022. We also searched ClinicalTrials.gov, the references of all included studies, conference proceedings, other systematic reviews and contacted experts in the field for unpublished data. Study selection, data extraction and quality assessment were performed independently and in duplicate by two review authors (L. K. and L. J.). Disagreements were resolved with the help of a third reviewer (J. A.).

### Eligibility criteria

We included randomized and quasi-randomized controlled trials examining the effects of burnout prevention programs in physicians of any medical speciality. The focus was on any form of burnout prevention, including both individual level (interventional) and organizational level interventions. We excluded cluster-randomized trials due to their increased susceptibility, particularly in this context, to selection bias, contamination, baseline imbalances and reporting bias. The control group received either no intervention or any other burnout prevention program. The outcome measure of interest was the prevalence of burnout in the participants as assessed by the authors of the respective study. Typically, this includes questionnaires on the severity of exhaustion, depersonalization, performance at work or attitude towards work. For trials with more than one time point of outcome assessment, we included the final assessment. Trials with no extractable data were included for the qualitative review if attempts to retrieve data from the authors were unsuccessful.

### Data extraction and risk of bias

A data extraction form was developed and piloted for this review. The following information was extracted for this review: types of studies, methods (randomization, handling of participant drop-out), types of participants and characteristics, setting of the studies, the form of the intervention, accompanying therapies and medication, outcome measures and adverse events, and comorbidities of the participants. We used the Cochrane risk of bias tool to assess the quality of the evidence [16], rating the risk of bias for each study as “low”, “unclear” or “high”.

### Statistical analysis

For the treatment effects, we performed meta-analyses using the inverse variance method, calculating the mean differences (MD) and 95% confidence intervals (CI). If trials used different outcome rating scales, the standardized mean difference was calculated to assess the treatment effect [15]. If standard deviations could not be retrieved, the averaged standard deviation of the other included trials was used as a proxy. If statistical heterogeneity was substantial, effect sizes were pooled using random-effects models. We assessed clinical heterogeneity and performed subgroup analyses to investigate the influence of relevant factors. We assessed funnel plot asymmetry visually and tested for publication bias [17]. In sensitivity analyses, we assessed whether study quality (e.g. high dropout rates) and studies causing heterogeneity (see Supplementary Appendix) influenced the main result.

## Results

### Results of the search

**Fig. 1:**
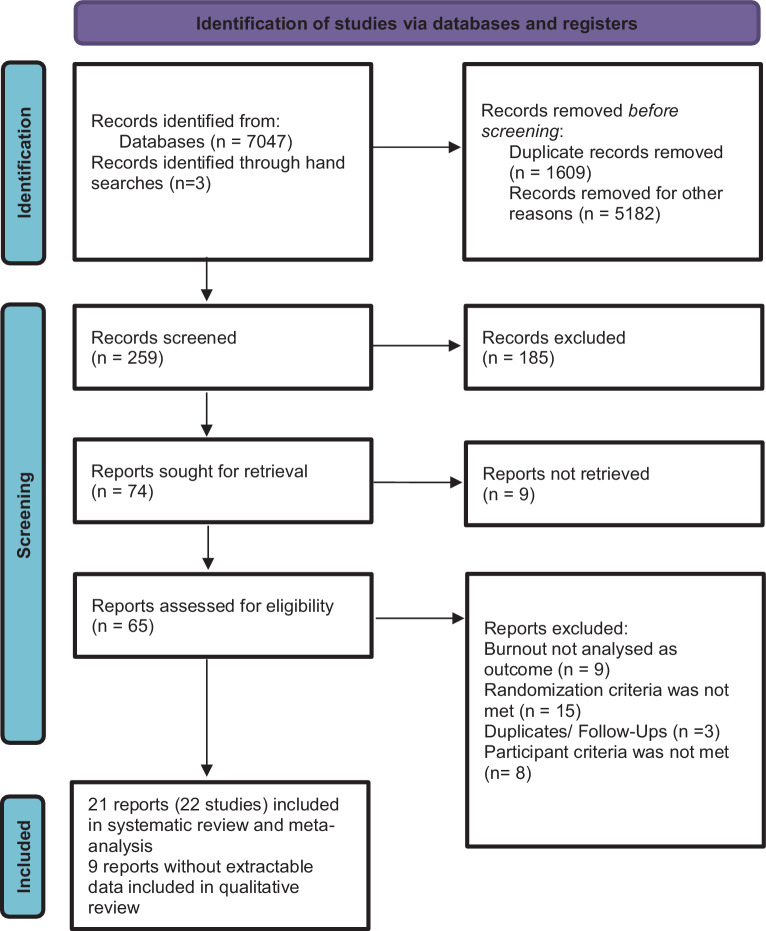
Study selection 2022

We retrieved 7074 titles and abstracts. After inspecting titles and abstracts, 21 studies were finally included in our systematic review (see Fig. 1).

Characteristics of the included trials can be found in Table 1. The number of participants varied from 21 to 279; in most trials, the majority of participants were female and 1 trial exclusively included female surgeons. Only two trials assessed structural approaches for burnout prevention intervention [18, 19]; all others evaluated individual level approaches. Some interventions took place in the work environment, others in retreats and in some studies interventions were offered online. The intervention period ranged between 1 day and 8 weeks. Mindfulness-based stress reduction (MBSR) or similar programs were the most common interventions but there were also programs using communication skills, role play, Balint groups, short-term psychotherapy or text message interventions. Most trials included participants from one specific medical field, such as pediatrics or oncology or included practising physicians from all fields. Participants of all included trials completed a questionnaire assessing baseline characteristics and the burnout prevalence. Most studies used the Maslach Burnout Inventory (MBI) or an abridged version, but only the subscale “emotional exhaustion” was used in all studies and therefore included in the analysis. Of the studies three used the Copenhagen Burnout Inventory, one used the Professional Quality of Life Scale and one trial used a Burnout subitem of the Personal Fulfilment Index (PFI). Shea et al. [20] included data from two locations, but because physicians rotated between the sites, we used the data from one location to avoid duplication. Parshuram et al. [18] compared different duty schedules (24, 16 and 12‑h schedules). To use their data, we treated the 24‑h schedule described as standard by the study authors as the control group and compared the other two groups against it. To avoid duplication, we divided the number of control group participants equally between the two comparisons.

**Table 1:** Characteristics of the included studies

Study ID	Characteristics of participants	Intervention	Control	Outcome
Amutio 2015 [21]	42 participants, practising physicians in various fields, 57% female	Mindfulness-based stress reduction, individual intervention	No intervention	MBI
Axisa 2019 [22]	46 participants, physician trainees in adult internal medicine and pediatrics, 74% female	Workshop about well-being, group work activities	No intervention	Professional Quality of Life Scale (ProQOL)
Bragard 2009 [23]	96 participants, oncology residents, 63% female	Communication skills training, stress management skills training, role plays	No intervention	MBI
Bragard 2010 [24]	62 participants, specialists working in cancer care, no statistically significant gender difference (no further information)	Communication skills training, stress management skills training, role plays	Baseline communication skills training, but without a consolidation workshop	MBI
Fendel 2021 [25]	147 participants, resident physicians in various fields, 65% female	Program based on the Mindfulness-Based Stress Reduction program	Active control: same coursebook, but no practical exercises	Copenhagen Burnout Inventory
Brazier 2021 [26]	279 participants, anesthetists in training, 52% female	Text message intervention	No intervention	Copenhagen Burnout Inventory
Gunasingam 2015 [27]	31 participants, postgraduate year 1 doctors on emergency, medical and surgical rotations, 47% female	Debriefing sessions	No intervention	MBI, SD missing
Huang 2020 [28]	36 participants, year‑1 residents in various fields, 44% female	Balint groups	No intervention	MBI
Ireland 2017 [29]	44 participants, intern doctors in emergency department rotation, 64% female	Mindfulness-based stress reduction	Active control group: 1 extra hour of break per week	Copenhagen Burnout Inventory
Lebares 2019 [30]	21 participants, postgraduate year‑1 surgery residents, 38% female	Modified Mindfulness-based stress reduction	Active control group: home practice requirements, retreat-hike format, weekly discussions	MBI
Lee 2020 [31]	243 participants, physicians working in polyclinics, 70% female	Short-term psychotherapy (Asimov method) based on a coping strategy	No intervention	MBI
Mache 2016 [32]	78 participants, junior gynecologists, 70% female	Coping skills training, discussion groups	No intervention	MBI
Mache 2017 [33]	80 participants, physicians in oncology and hematology, 59% female	Psychosocial competency training combined with cognitive behavioral and solution-focused counselling	No intervention	MBI
Martins 2011 [34]	74 participants, pediatric residents, 83% female	Workshops on burnout, risk factors and coping strategies	No intervention	MBI, SD missing
McGonagle 2020 [35]	59 participants, primary care physicians, less than 25 years of work experience, 79% female	Coaching intervention (6 coaching sessions)	No intervention	MBI
Palamara 2022 [36]	237 surgery residents, 100% female	Coaching intervention	Informational material related to physician well-being	PFI subscale for burnout
Parshuram 2015 [18]	47 ICU residents (internal medicine, anesthesia, surgery, emergency medicine), no information on gender disparity	12-h/16‑h shift model on ICU, structural approach	Regular shift model (24 h)	MBI
Salyers 2019 [37]	192 participants, clinicians at mental health centers, 80% female	Burnout reduction program: BREATHE (enhanced awareness, tools, handouts and education)	Active control: motivational interviewing	MBI
Shea 2014 [19]	106 internal medicine interns, 52% female	Protected sleep period on overnight call shifts, structural approach	No intervention	MBI
Schroeder 2016 [20]	33 participants, primary care physicians, no significant difference in demographic values (no further information)	Modified mindfulness-based stress reduction	No intervention	MBI
Verweij 2018 [38]	138 participants, residents from various disciplines, 88% female	Mindfulness-based stress reduction	No intervention	MBI

*MBI* Maslach Burnout Inventory, *ProQOL* Professional Quality of Life Scale, *SD* standard deviation, *PFI* Personal Fulfilment Index

### Risk of bias in included studies

The details and justifications of our risk of bias assessment are listed in the Supplementary Appendix. Generally, for all studies there was some concern about bias and eight studies had a high risk of bias [20, 24, 26, 31, 33, 37, 39, 40]. Most high-risk of bias judgements were a result of incomplete outcome reporting, eight trials did not provide more information on the random sequence generation [18–20, 24, 28, 29, 34, 40] and none of the studies gave details on blinding of the outcome assessors.

### Main outcomes

**Fig. 2:**
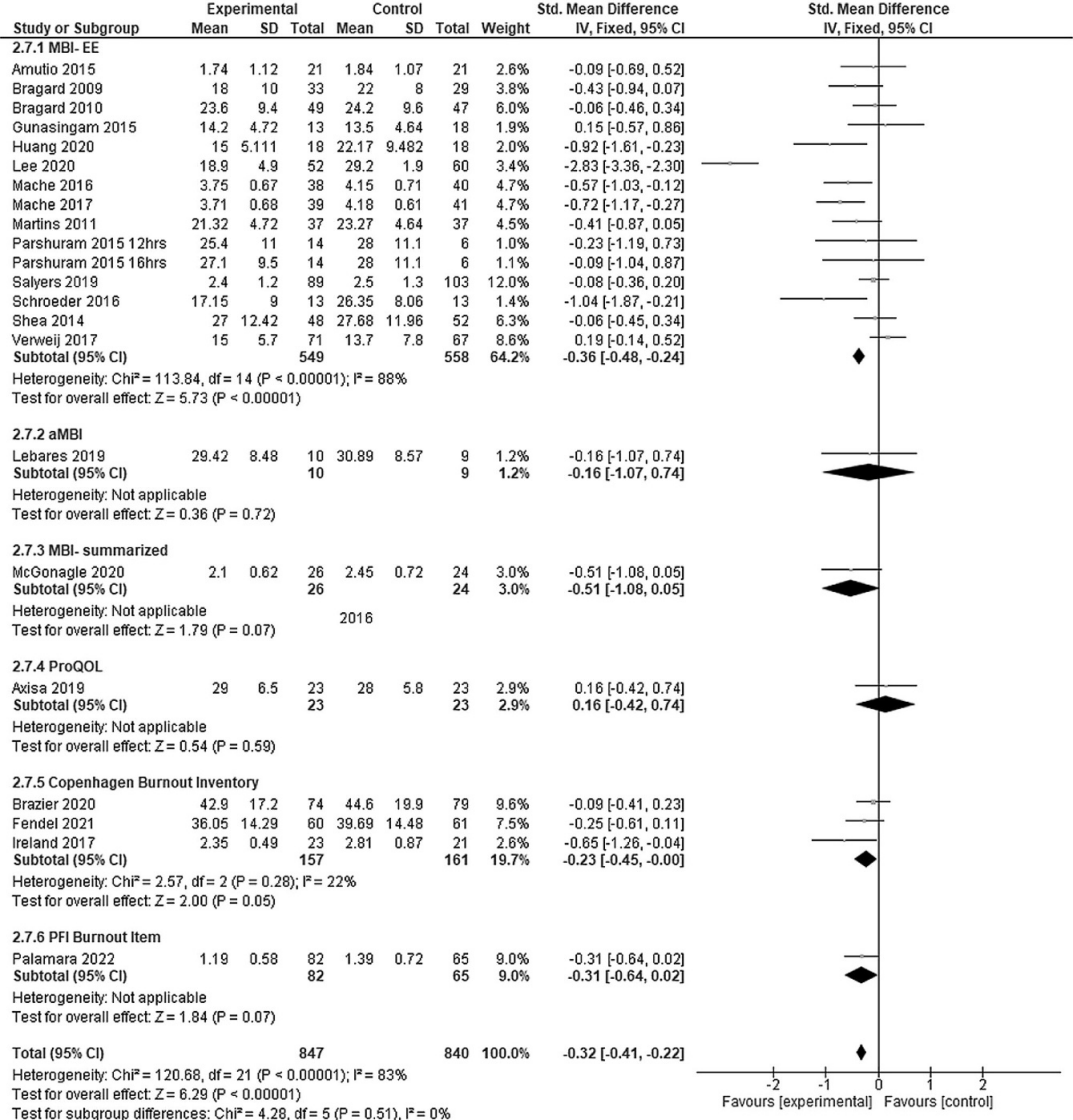
Results of the main analysis. *MBI-EE* Maslach Burnout Inventory, dimension of emotional exhaustion, *aMBI* abbreviated Maslach Burnout Inventory, *MBI-summarized* Maslach Burnout Inventory-summarized, *ProQOL* Professional Quality of Life scale. The index numbers are automatically generated by the software and have no further relevance

Overall, the use of burnout prevention programs resulted in a significant reduction of burnout when compared to the control groups by an SMD of −0.32 (95% CI: −0.41 to −0.22; see Forest plot Fig. 2), which may be interpreted as a small effect [15].

**Fig. 3:**
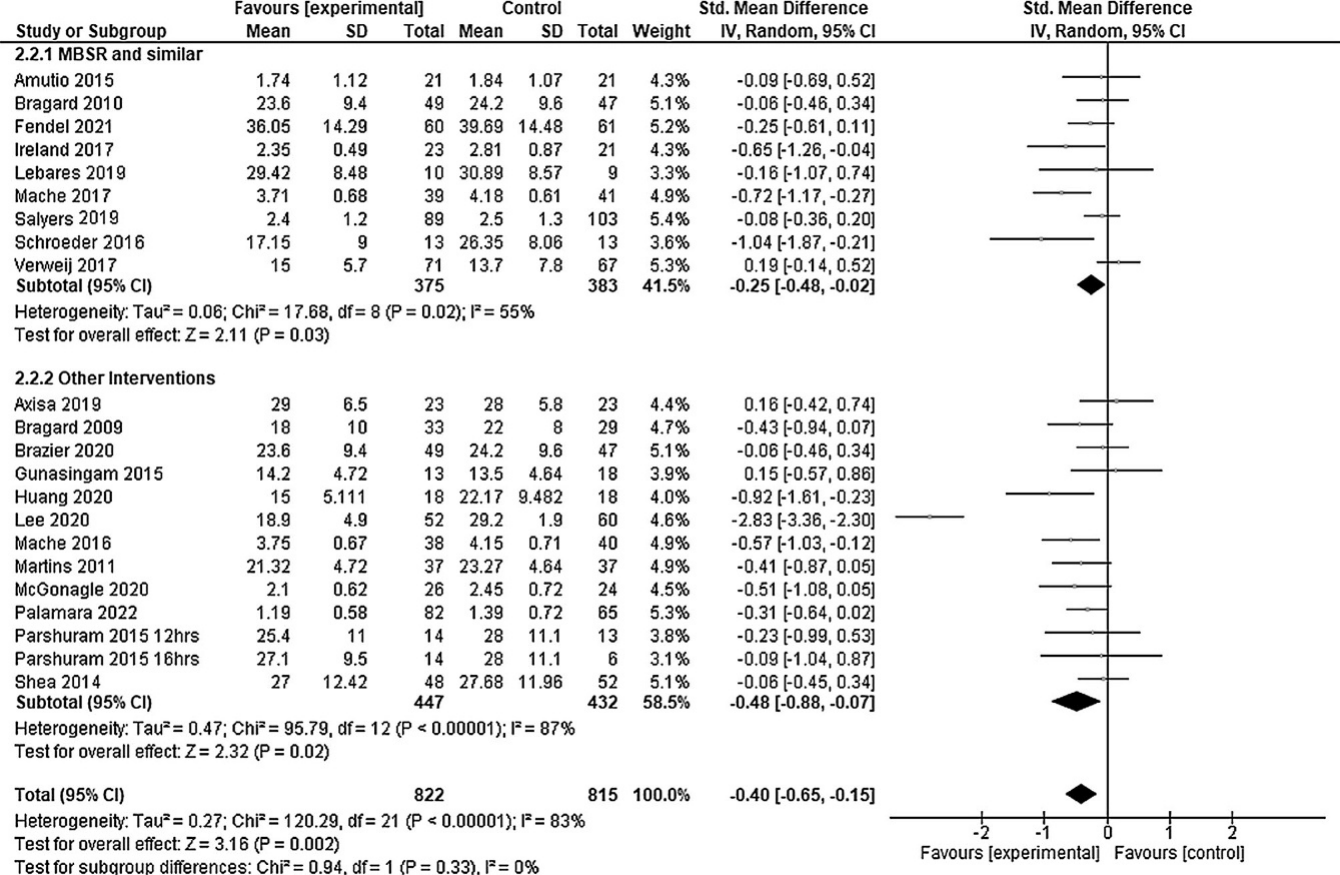
Subgroup analysis mindfulness-based stress reduction (MBSR) versus other programs

We performed subgroup analyses according to comparable burnout intervention programs. The MBSR and programs similar in structure and length were used in about half of all studies. The subgroup analysis showed a significant reduction in burnout in the group performing MBSR or similar interventions SMD −0.25 (95% CI: −0.48 to −0.02) as well as in the group performing other interventions SMD −0.48 (95% CI: −0.88 to −0.07) (see Fig. 3), the effect of both subgroups can be interpreted as small. Confidence intervals of both subgroups were largely overlapping. The index numbers are automatically generated by the software and have no further relevance.

**Fig. 4:**
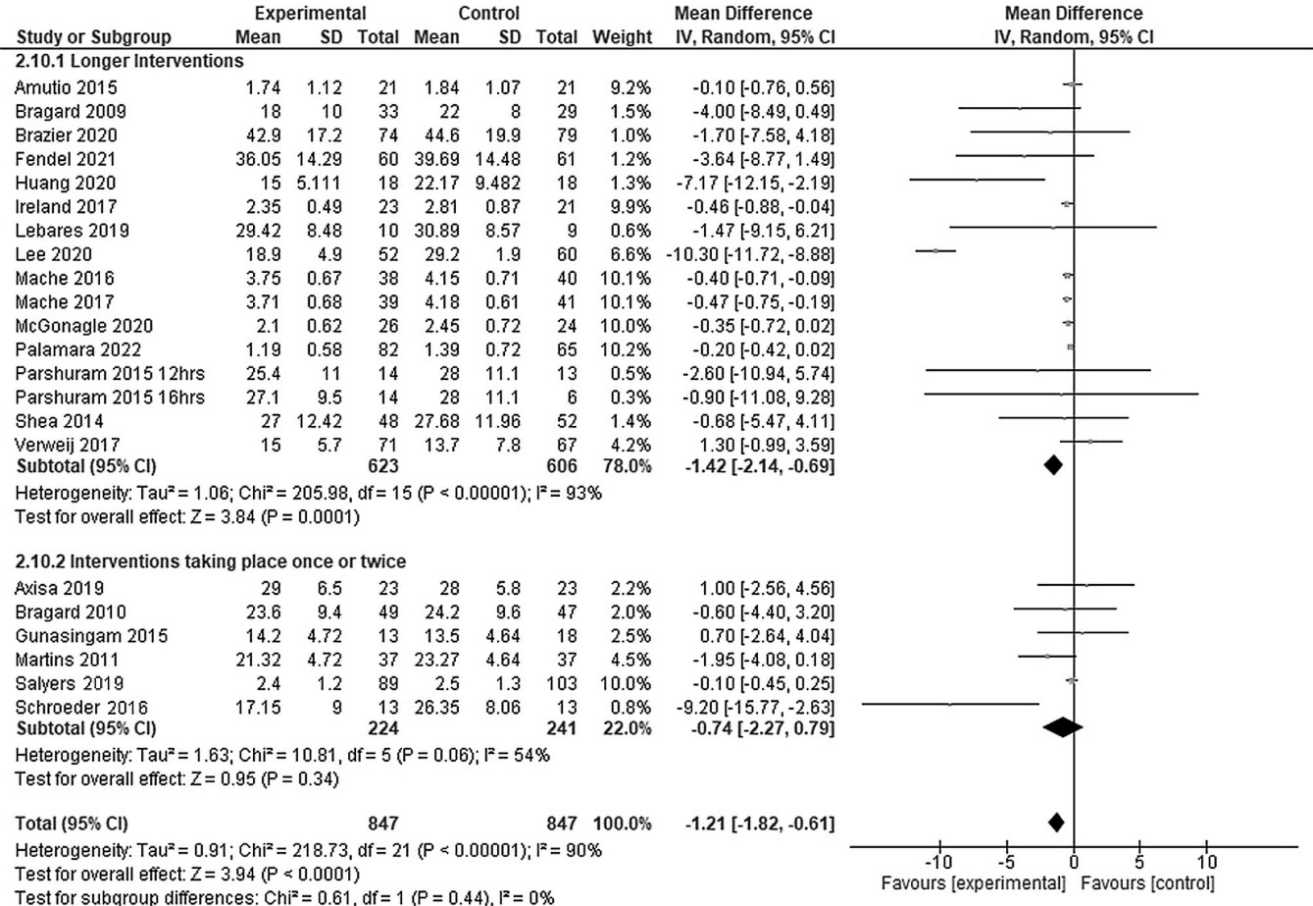
Longer interventions versus interventions that took place only once or twice

The subgroup analysis according to the length of the intervention (see Fig. 4) showed that prevention programs that lasted over several weeks had a much higher effect than the summary result SMD −1.42 (95% CI: −2.14 to −0.69), while interventions that took place only once or twice did not show a significant effect SMD −0.74 (95% CI: −2.27 to 0.79). The index numbers are automatically generated by the software and have no further relevance.

**Fig. 5:**
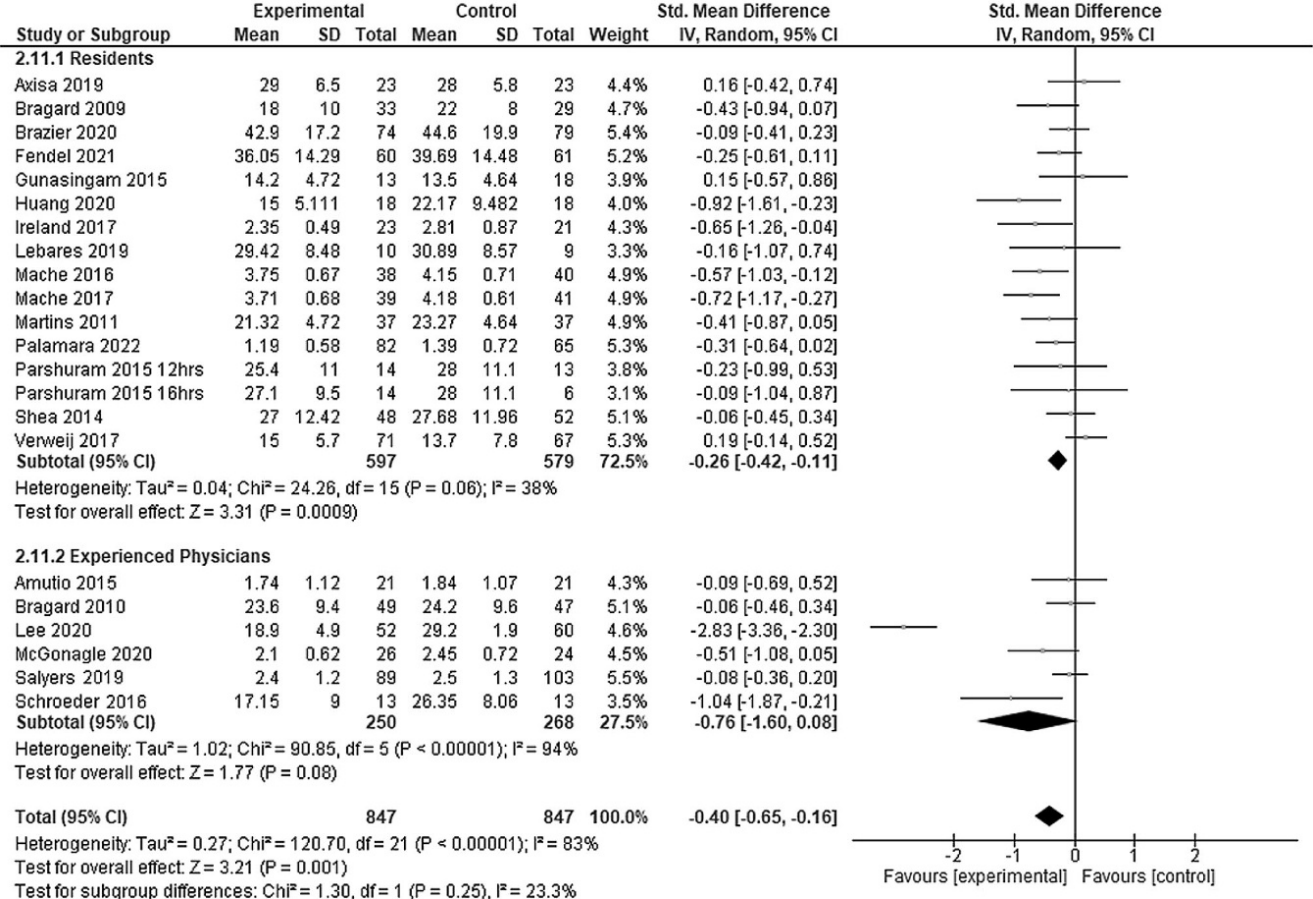
Residents versus experienced physicians as participants

In the subgroup analysis according to seniority of the study participants (residents versus experienced physicians, see Fig. 5) we observed a smaller effect size in the group of resident participants SMD −0.26 (95% CI: −0.42 to −0.11) than for the more senior physicians SMD −0.76 (95% CI: −1.60 to 0.08). The index numbers are automatically generated by the software and have no further relevance.

### Sensitivity analyses

**Fig. 6:**
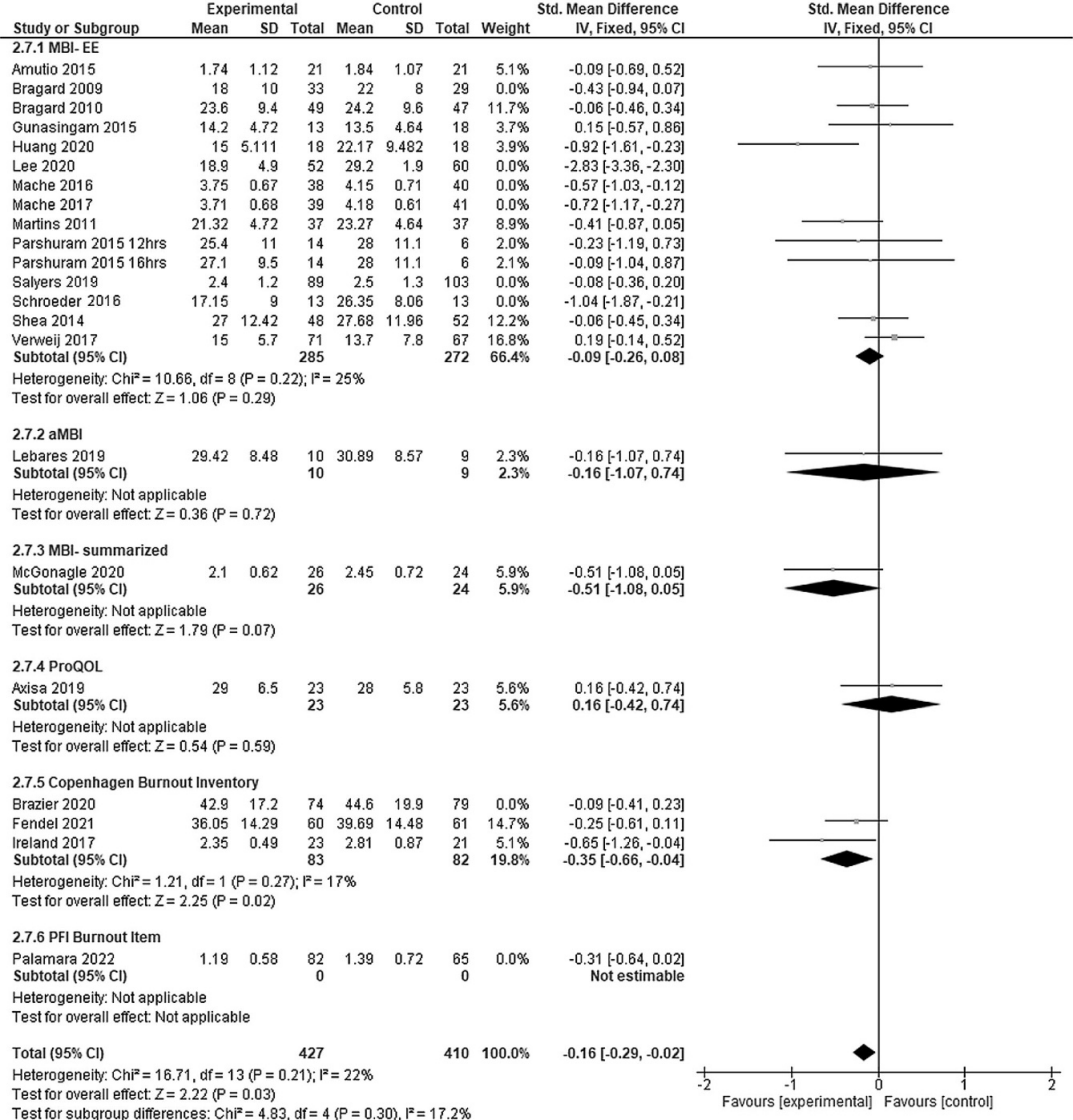
Sensitivity analysis: excluding trials with high risk of bias in at least one domain. *MBI-EE* Maslach Burnout Inventory, dimension of emotional exhaustion, *aMBI* abbreviated Maslach Burnout Inventory, *MBI-summarised* Maslach Burnout Inventory-summarized, *ProQOL* Professional Quality of Life Scale

Of the trials eight had a high risk of bias in at least one of the domains, mostly because of high dropout rates (see Supplementary Appendix for risk of bias graph). When we excluded these studies in a sensitivity analysis (Fig. 6), the summary effect was reduced to −0.16 (95% CI: 0.29 to −0.02). The index numbers are automatically generated by the software and have no further relevance.

Heterogeneity was high in the main analysis (I^2^ 83%) and was mainly driven by one trial with a large treatment effect compared to the other trials (Lee et al. 2020) [31]. Excluding this trial in a sensitivity analysis substantially reduced heterogeneity between studies (I^2^ = 35%). Looking at clinical heterogeneity, we found two differentiating factors that set the study of Lee et al. apart. 1. The intervention was short-term psychotherapy (Asimov method) based on a coping strategy; no other program used psychotherapy as a prevention program. 2. The study was conducted in Kazakhstan, while most other trials were conducted in western countries. Country-specific healthcare and cultural factors may have influenced the effect of the burnout intervention. The index numbers are automatically generated by the software and have no further relevance.

### Publication bias

**Fig. 7:**
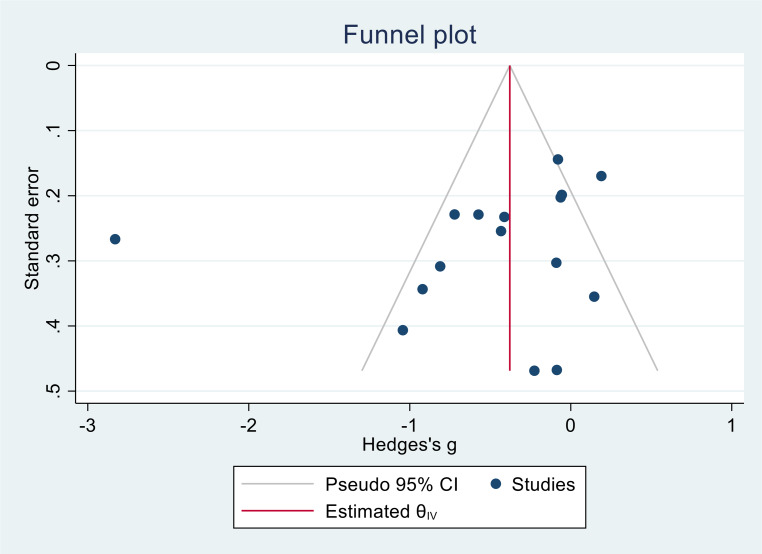
Funnel plot analysis with the highest number of studies (emotional exhaustion), the Egger test showed little evidence for small study effects (*p* = 0.6)

Funnel plots of the main analysis did not suggest publication bias (see Fig. 7). The Egger test showed little evidence for small study effects (*p* = 0.6).

## Discussion

Results from this systematic review and meta-analysis, including 22 studies with 1687 participants, showed that burnout prevention programs were only marginally effective in reducing the burnout prevalence among practising physicians. The overall small to moderate effect was reduced even further after the sensitivity analysis, excluding studies with a high risk of bias. Heterogeneity between the included studies was high, and after excluding the one study causing the most heterogeneity, the effect was mitigated.

Beyond the finding that individual burnout prevention programs seem to have small health effects, we also identified valuable insights, for example, interventions lasting several weeks or more tended to have a greater effect on burnout prevention than shorter programs. Another important finding reflects the overall low quality of studies in the field. Of 22 included studies, 8 had a risk of bias classified as “high” in one domain (see Supplementary Appendix Fig. 4), mainly caused by incomplete outcome reporting.

### Limitations and strengths in the review process

Of the studies nine did not provide data in an extractable format (e.g. change from baseline as the only result) and despite several attempts the study authors could not be reached for further information. These studies were included in the qualitative review (see Supplementary Appendix table 2). Their findings largely corresponded with the meta-analysis, as most interventions had a small effect on the prevention of burnout.

We found that the risk of bias was mainly due to incomplete outcome reporting. Burnout research may be especially susceptible to methodological challenges such as selection bias and participant attrition. The most vulnerable physicians may be less likely to participate and most likely to drop out of the study. Real-world variables such as staffing shortages and changes in workload during the intervention period can further limit the generalizability of burnout prevention trials and need to be taken into consideration.

Another limitation was the limited information on the work environment of the participating physicians (such as ward work, outpatient clinic or acute care). This may be important as different work environments may expose physicians to varying stressors. At a reviewer’s request, we looked deeper into this matter and conducted a post hoc analysis with physicians in acute settings like emergency medicine, polyclinics, primary care [20, 29, 31, 35]. The effect was more pronounced and statistically significant SMD −1.27 (95% CI: −2.45 to −0.08). While this subgroup analysis should be interpreted with caution, it may indicate that burnout prevention programs are more effective in acute care settings. Generally, there was a substantial variety of interventions, which we tried to accommodate with subgroup analyses. Most studies showed no effect or a small treatment effect, suggesting that the true effect may also be located in this range.

Even if trials used the same questionnaire, as the Maslach Burnout Inventory, many authors used different versions and not all dimensions. “Emotional exhaustion” was the dimension of the MBI that was reported in all studies and, therefore, most suitable as the main outcome parameter. Including all three dimensions of the MBI would have resulted in an overestimation of the effect. In our sensitivity analyses; however, we could show that if we had chosen another dimension like “personal accomplishment” (PA) or “depersonalization” (DP), the results would not have meaningfully changed (see Supplementary Appendix Figures 2, 3).

Maslach et al. addressed those misuses of the MBI and argued against a simplification of the questionnaire [41]. The possibility that burnout instruments, while validated may lack sensitivity to detect subtle changes should be acknowledged, as variation in subscale use and scoring may have further limited the ability to capture meaningful effects. For further research in this field, it would be advisable to standardize the assessment of burnout severity. Other reviews show that most interventions succeeded in lowering the rates of burnout in the experimental group of those trials, mostly through some form of mindfulness courses [10]; however, they did not show whether one burnout prevention program is especially suitable for preventing burnout in physicians or if individual approaches are more or less successful.

Another finding of this review is that there could be improvements in burnout research when focusing on the most promising burnout intervention programs. When trying to find explanations for the small effect, the burnout literature offers explanations by pointing out that burnout seems to be a structural, rather than an individual problem in healthcare systems, suggesting that an organizational prevention approach may be more effective [42]. West et al. explained that factors like high workload, long working hours, the increase of documentation tasks and frequent overnight or weekend shifts contribute to physician burnout [43]. Other reviews advocated individual solutions, such as psychosocial interventions [44].

This review and meta-analysis found only two trials which assessed a structural intervention [18, 19]. Parshuram et al. [18] compared different duty schedules (24, 16 and 12‑h schedules) among ICU residents, while Shea et al. [19] implemented 5 h of protected sleep in overnight call shifts for interns of internal medicine. Neither individually nor combined in a post hoc subgroup analysis SMD −0.08 (95% CI: −0.42 to 0.26), the study showed a significant effect of the intervention but may have been too small to detect meaningful differences.

Other studies, however, emphasize that the factors contributing to physician burnout are primarily structural. The treatment and prevention of burnout should not only include teaching tools to enhance individual resilience but also address the underlying systemic causes of burnout [45, 46]. Emotional exhaustion of participating physicians correlated with the number of cancer patients treated [24] or the increase in clinical practice [24]. A new trial by Fang et al. showed that with the rising number of working hours, the rates of depression in U.S. first-year physicians increased as well [47]. Other reported risks were difficulty in maintaining work-life balance, lack of support from senior staff and inadequate supervision [27].

A very recent multinational European Delphi Survey, initiated by the European Society for Emergency Medicine (EUSEM), rated burnout, exhaustion, and mental fatigue as the most important adverse personal health events. The authors found that even in varying healthcare systems, improvement strategies should focus on system-related external stressors [48].

Medical bureaucracy seems to play another major role in the burnout of healthcare providers. Studies show that they spend at least as much time documenting as with direct patient care [49] and that the time spent on administration tasks correlates with low career satisfaction [50]. Taking those factors into account, it seems that the widespread burnout prevention programs, as well as current research, are not focusing on the most likely causes of burnout.

## Conclusion

Burnout remains one of the most relevant diseases affecting healthcare workers all over the world. Various studies pointed out how it affects not only the mental but also the physical health of the person as well as patient safety.

This systematic review and meta-analysis showed that different prevention programs addressing mindfulness have a very limited effect on reducing the burnout prevalence in physicians, and other strategies, focusing on the sources of burnout, may be more promising. This may include structural changes or individual approaches that address the specific needs of each physician.

## Supplementary Information

Supplementary appendix
