# Supplementary material for: Prevention of burnout syndrome in physicians: a systematic review and meta-analysis
Source: Wien Klin Wochenschr. 2025 Nov 10;138(5-6):167–78. doi: 10.1007/s00508-025-02601-y (PMC12992466; doi:10.1007/s00508-025-02601-y)
Supplement: Supplementary file 1 — Supplementary appendix [file 508_2025_2601_MOESM1_ESM.docx]

**Supplementary appendix**

**1. Sensitivity Analysis**


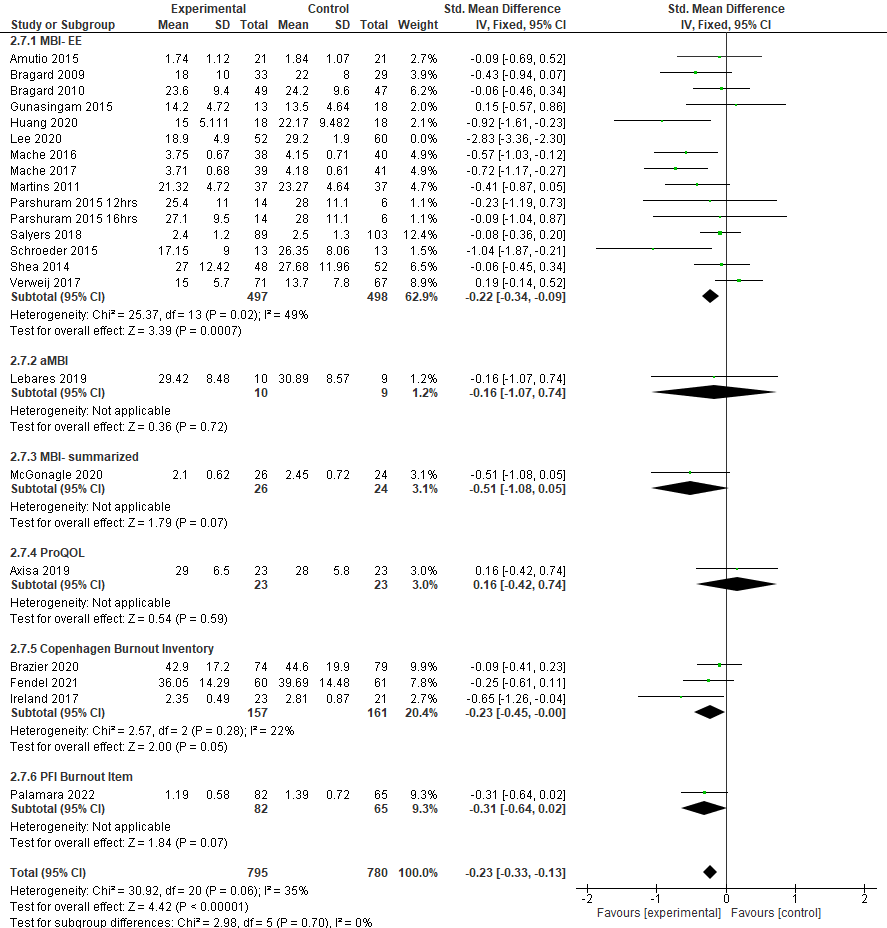


Supplementary appendix Figure 1: Sensitivity Analysis: excluding Lee 2020 (MBI-EE: Maslach Burnout Inventory, dimension of Emotional Exhaustion, aMBI: abbreviated Maslach Burnout Inventory, MBI-summarized: Maslach Burnout Inventory- summarized, ProQOL: Professional Quality of Life Scale), PFI- Personal Fulfillment Index


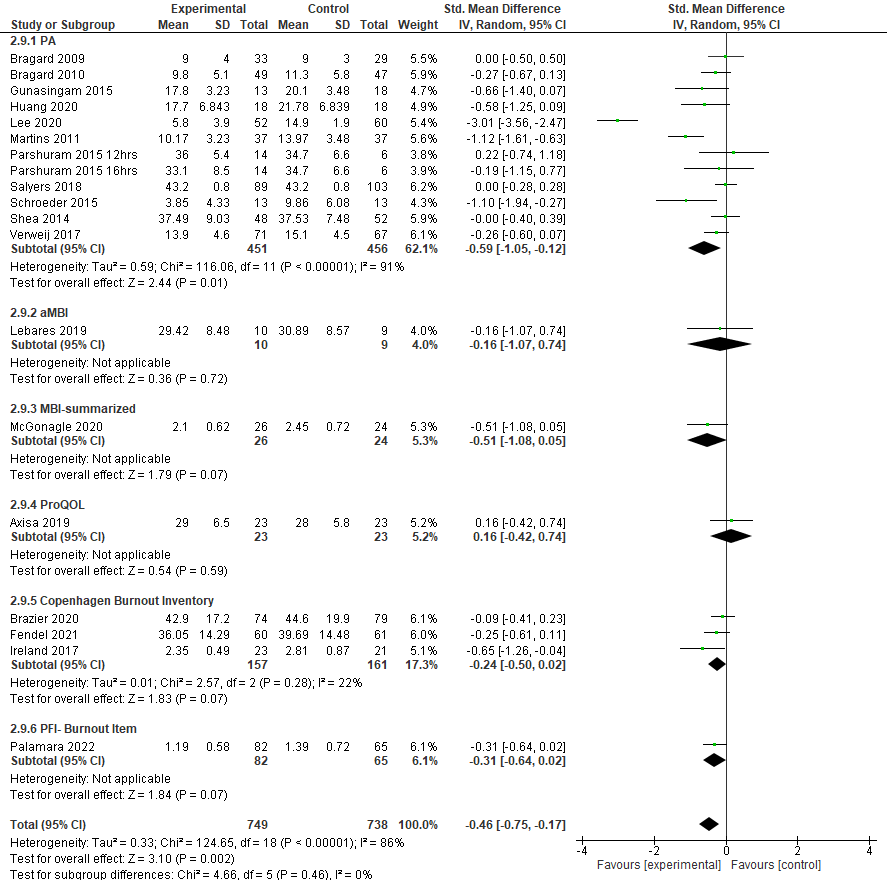


Supplementary appendix Figure 2: main analysis using another subset of the MBI (PA) (PA: Maslach Burnout Inventory, dimension of Personal Accomplishment, aMBI: abbreviated Maslach Burnout Inventory, MBI-summarised: Maslach Burnout Inventory- summarised, ProQOL: Professional Quality of Life Scale), PFI- Personal Fulfilment Index


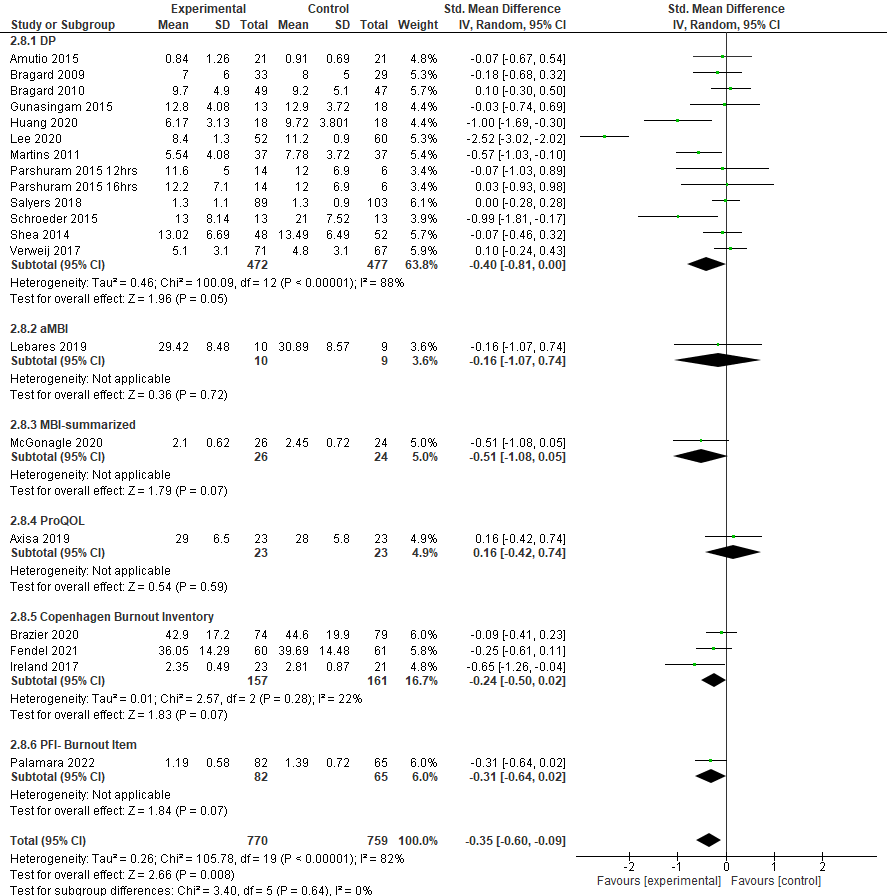


Supplementary appendix Figure 3: main analysis using another subset of the MBI (DP) (DP: Maslach Burnout Inventory, dimension of Depersonalization, aMBI: abbreviated Maslach Burnout Inventory, MBI-summarised: Maslach Burnout Inventory- summarised, ProQOL: Professional Quality of Life Scale), PFI- Personal Fulfilment Index

**2. Detailed Search Strategy:**

| Medline/ PsycINFO/ CENTRAL/ BIOSIS/ SCOPUS | (burnout OR emotional exhaustion OR occupational stress) AND (physician* or doctor* OR medic* OR intern* OR residen* OR surgeon* OR medical staff OR emergency medicine OR internal medicine OR anesthesiology) AND (intervention* OR program* OR strateg*)  Randomised controlled trial filter as provided by the databases |
| --- | --- |

Supplementary Appendix Table 1

**3. Risk of Bias in included trials**

**Randomisation and Allocation Concealment**

In 8 out of 21 trials, the randomisation process was considered “unclear” in the risk of bias; others were considered to have a low risk of bias. Only 5 trials reported their methods of allocation concealment (1-5).

**Blinding**

Due to the nature of the interventions, blinding of participants and personnel was not possible in any of the included trials.

**Incomplete outcome data**

Five trials (6-10) had dropout rates over 20%, which we considered to be large according to the ‘Risk of bias’ tool.

**Selective reporting**

There were no indications that the reporting of data had been selected for most included trials. In most cases, study protocols or trial registrations were missing, which led us to consider the bias as “unclear”. In two cases, only one domain of the outcome assessment was reported, which led us to consider the risk of bias in those cases as “high” (4, 11).

**Other potential sources of bias**

In many trials, there were significant baseline differences in the sex of the participants, with, in many cases, more female participants (see Figure 2).


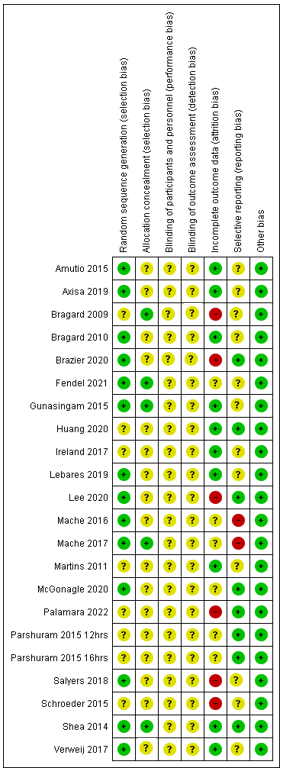


Supplementary appendix Figure 4: Risk of bias in included studies

**4. Summary of trials excluded due to missing data (qualitative review)**

Gabbe 2008 (12) conducted an intervention of mentoring for new chairs of obstetrics and gynaecology and assessed how stress and burnout rates were affected. After one year of mentoring, no difference were observed in burnout rates between the experimental and control group. Lebares 2021 (13) examined two different burnout prevention programs that were based on the MBSR course, which were called ESRT-1 and -2. ESRT-1 was a course teaching mindfulness meditation skills in a time frame of 8 weeks with weekly 2-hour sessions and was directed at physicians of all specialities. ESRT-2 focused on a more practical and surgery-focused approach of mindfulness and involved 6 weekly 90-minute classes. Both were compared to two active control groups, which comprised classes equal in time and setting to the intervention groups, with briefings about stressful aspects of medical training and discussions on general work-related topics. Burnout, measured through the 9-item MBI, was not significantly different for the ESRT-1 group. In the ESRT-2 group (using the 2-item MBI), “emotional exhaustion” and “depersonalization” were significantly lower after the intervention compared to their control group.
Fainstad 2022 (14) analysed whether an online group-coaching program would reduce burnout rates in female residents. Emotional exhaustion was reduced by the end of the trial in the experimental group versus the control group. In the Congiusta 2020 (15)trial, a 24-week online wellbeing program was the performed intervention. It showed a significant reduction of physician burnout in the intervention group.
Loewenthal 2021 (16) examined whether Yoga could reduce physician stress and burnout. They found that mindfulness, stress and burnout rates improved after the intervention.
Dyrbye 2016 (17) performed an online intervention on job satisfaction and wellbeing as well. After the intervention took place, the experimental group did improve in fatigue and job satisfaction. The burnout dimensions, however, did not change significantly compared to the control group. The Ripp 2016 (18) trial examined if theme- based discussion sessions were effective in reducing burnout. After the intervention took place, burnout had not decreased in the participating physicians.
West 2014 (19) also performed physician discussion groups. The groups incorporated themes like mindfulness, reflection and shared experience. Burnout rates sank significantly in the intervention group. Dyrbye 2019 (20) investigated whether coaching interventions on well-being would reduce burnout rates in physicians. They found that emotional exhaustion decreased significantly in the intervention group, whereas there was no significant difference in depersonalization.

| **Study ID** | **Intervention** | **Characteristics of participants** | **Control** | **Outcome** |
| --- | --- | --- | --- | --- |
| Gabbe 2008 | Mentoring by experienced chairs of gynaecology | 17 participants, new gynaecology chairs, 22% female | No intervention | MBI |
| Lebares 2021 | Tailored Mindfulness-based Stress Reduction program | 89 participants, resident trainees and first-year surgical trainees, gender distribution not stated | Active control group: group reading, protected time | Modified MBI |
| Fainstad 2022 | Web-based group-coaching program | 101 participants, resident physicians, 100% female | No Coaching | MBI |
| Congiusta 2020 | Online training program | 63 physicians from different disciplines, 38% female | No Intervention | MBI |
| Loewenthal 2021 | Yoga-based mind-body intervention | 44 participants, resident physicians, 82% female | No Intervention | 2-item-MBI |
| Dyrbye 2016 | Weekly micro-tasks on well-being | 290 participants, practising physicians, 30% female | No intervention | MBI |
| Ripp 2016 | Theme-based discussion sessions | 51 participants, incoming internal medicine trainees, gender distribution not stated | No intervention | MBI |
| West 2014 | Physician discussion groups | 74 participants, practising physicians, 34% women | 1 hour of protected time per week | MBI |
| Dyrbye 2019 | Coaching sessions | 88 participants, practising physicians, 55% women | No intervention | MBI |

Supplementary appendix Table 2: Summary of results of studies with missing data (qualitative review)

1. Bragard I, Etienne AM, Merckaert I, Libert Y, Razavi D. Efficacy of a communication and stress management training on medical residents' self-efficacy, stress to communicate and burnout: a randomized controlled study. J Health Psychol. 2010;15(7):1075-81. doi:10.1177/1359105310361992

2. Fendel JC, Aeschbach VM, Schmidt S, Goritz AS. The impact of a tailored mindfulness-based program for resident physicians on distress and the quality of care: A randomised controlled trial. Journal of internal medicine. 2021;290(6):1233-48. doi:<https://dx.doi.org/10.1111/joim.13374>

3. Gunasingam N, Burns K, Edwards J, Dinh M, Walton M. Reducing stress and burnout in junior doctors: the impact of debriefing sessions. Postgrad Med J. 2015;91(1074):182-7. doi:10.1136/postgradmedj-2014-132847

4. Mache S, Vitzthum K, Hauschild I, Groneberg D. A pilot study evaluation of psychosocial competency training for junior physicians working in oncology and hematology. Psychooncology. 2017;26(11):1894-900. doi:10.1002/pon.4403

5. Shea JA, Bellini LM, Dinges DF, Curtis ML, Tao Y, Zhu J, et al. Impact of protected sleep period for internal medicine interns on overnight call on depression, burnout, and empathy. J Grad Med Educ. 2014;6(2):256-63. doi:10.4300/JGME-D-13-00241.1

6. Salyers MP, Garabrant JM, Luther L, Henry N, Fukui S, Shimp D, et al. A Comparative Effectiveness Trial to Reduce Burnout and Improve Quality of Care. Adm Policy Ment Health. 2019;46(2):238-54.

7. Brazier A, Larson E, Frerichs J, Milward J, Judah G, Darzi A. “Dear Doctor” text message intervention to reduce burnout in trainee anaesthetists: an interview study. Lancet. 2021;398:S26‐. doi:10.1016/S0140-6736(21)02569-1

8. Lee S, Rozybakieva Z, Asimov M, Bagiyarova F, Tazhiyeva A, Ussebayeva N, et al. Coping strategy as a way to prevent emotional burnout in primary care doctors: A randomized controlled trial. Archives of the Balkan Medical Union. 2020;55(3):398-409. doi:10.31688/ABMU.2020.55.3.05

9. Bragard I, Libert Y, Etienne AM, Merckaert I, Delvaux N, Marchal S, et al. Insight on variables leading to burnout in cancer physicians. J Cancer Educ. 2010;25(1):109-15. doi:10.1007/s13187-009-0026-9

10. Schroeder DA, Stephens E, Colgan D, Hunsinger M, Rubin D, Christopher MS. A Brief Mindfulness-Based Intervention for Primary Care Physicians: A Pilot Randomized Controlled Trial. Am J Lifestyle Med. 2016;12(1):83-91. doi:10.1177/1559827616629121

11. Mache S, Baresi L, Bernburg M, Vitzthum K, Groneberg D. Being prepared to work in Gynecology Medicine: evaluation of an intervention to promote junior gynecologists professionalism, mental health and job satisfaction. Arch Gynecol Obstet. 2017;295(1):153-62. doi:10.1007/s00404-016-4223-6

12. Gabbe SG, Webb LE, Moore DE, Mandel LS, Melville JL, Spickard WA. Can mentors prevent and reduce burnout in new chairs of departments of obstetrics and gynecology: results from a prospective, randomized pilot study. Am J Obstet Gynecol. 2008;198(6):e1-7. doi:ARTN 653.e1

10.1016/j.ajog.2007.11.004

13. Lebares CC, Coaston TN, Delucchi KL, Guvva EV, Shen WT, Staffaroni AM, et al. Enhanced Stress Resilience Training in Surgeons: Iterative Adaptation and Biopsychosocial Effects in 2 Small Randomized Trials. Annals of surgery. 2021;273(3):424‐32. doi:10.1097/SLA.0000000000004145

14. Fainstad T, Mann A, Suresh K, Shah P, Dieujuste N, Thurmon K, et al. Effect of a Novel Online Group-Coaching Program to Reduce Burnout in Female Resident Physicians: A Randomized Clinical Trial. JAMA network open. 2022;5(5):e2210752. doi:<https://dx.doi.org/10.1001/jamanetworkopen.2022.10752>

15. Congiusta S, Ascher EM, Ahn S, Nash IS. The Use of Online Physician Training Can Improve Patient Experience and Physician Burnout. American journal of medical quality : the official journal of the American College of Medical Quality. 2020;35(3):258-64. doi:<https://dx.doi.org/10.1177/1062860619869833>

16. Loewenthal J, Dyer NL, Lipsyc-Sharf M, Borden S, Mehta DH, Dusek JA, et al. Evaluation of a Yoga-Based Mind-Body Intervention for Resident Physicians: A Randomized Clinical Trial. Global Advances In Health and Medicine. 2021;10. doi:10.1177/21649561211001038

17. Dyrbye LN, West CP, Richards ML, Ross HJ, Satele D, Shanafelt TD. A randomized, controlled study of an online intervention to promote job satisfaction and well-being among physicians. Burnout research. 2016;3(3):69‐75. doi:10.1016/j.burn.2016.06.002

18. Ripp J, Fallar R, Korenstein DR. A Randomized Controlled Trial to Decrease Job Burnout in First-Year Internal Medicine Residents Using a Facilitated Discussion Group Intervention. Journal of General Internal Medicine. 2015;30:S90-S.

19. West CP, Dyrbye LN, Rabatin JT, Call TG, Davidson JH, Multari A, et al. Intervention to promote physician well-being, job satisfaction, and professionalism: a randomized clinical trial. JAMA internal medicine. 2014;174(4):527‐33. doi:10.1001/jamainternmed.2013.14387

20. Dyrbye LN, Shanafelt TD, Gill PR, Satele DV, West CP. Effect of a Professional Coaching Intervention on the Well-being and Distress of Physicians: A Pilot Randomized Clinical Trial. JAMA Internal Medicine. 2019;179(10):1406‐14. doi:10.1001/jamainternmed.2019.2425
